# Protein Phosphatase 1 Regulates Human Cytomegalovirus Protein Translation by Restraining AMPK Signaling

**DOI:** 10.3389/fmicb.2021.698603

**Published:** 2021-07-15

**Authors:** Carmen Stecher, Sanja Marinkov, Lucia Mayr-Harting, Ana Katic, Marie-Theres Kastner, Franz J. J. Rieder-Rommer, Xionghao Lin, Sergei Nekhai, Christoph Steininger

**Affiliations:** ^1^Division of Infectious Diseases and Tropical Medicine, Department of Medicine I, Medical University of Vienna, Vienna, Austria; ^2^Center for Sickle Cell Disease, Howard University, Washington, DC, United States

**Keywords:** PP1, HCMV, AMPK, phosphatase, tegument, eEF2

## Abstract

Human cytomegalovirus (HCMV) carries the human protein phosphatase 1 (PP1) and other human proteins important for protein translation in its tegument layer for a rapid supply upon infection. However, the biological relevance behind PP1 incorporation and its role during infection is unclear. Additionally, PP1 is a difficult molecular target due to its promiscuity and similarities between the catalytic domain of multiple phosphatases. In this study, we circumvented these shortcomings by using 1E7-03, a small molecule protein–protein interaction inhibitor, as a molecular tool of noncatalytic PP1 inhibition. 1E7-03 treatment of human fibroblasts severely impaired HCMV replication and viral protein translation. More specifically, PP1 inhibition led to the deregulation of metabolic signaling pathways starting at very early time points post-infection. This effect was at least partly mediated by the prevention of AMP-activated protein kinase dephosphorylation, leading to elongation factor 2 hyperphosphorylation and reduced translation rates. These findings reveal an important mechanism of PP1 for lytic HCMV infection.

## Introduction

The Ser/Thr protein phosphatase 1 (PP1) is conserved from yeast to humans and abundant in many cell types. PP1 is estimated to dephosphorylate the majority of Ser/Thr-linked phosphorylations on eukaryotic proteins (Heroes et al., 2013). This implies that PP1 counteracts hundreds of distinct Ser/Thr kinases, which is reflected in its functional diversity. Unsurprisingly, catalytic inhibition of PP1 and other phosphatases is highly toxic (Munday, 2013), making them challenging drug targets. However, PP1 exerts its functions as a holoenzyme composed of a catalytic subunit complexed with one of over 200 recognized regulatory proteins (PP1R), interacting with PP1 via docking motifs such as the RVxF, SpiDoc, SILK, IDoHA, or MyPhonE motifs (Shi, 2009; Rebelo et al., 2015). Several viruses exploit this regulatory mechanism by expressing viral PP1Rs to benefit their own replication. Specific viral PP1 targeting proteins promote eIF2α dephosphorylation, such as Herpes simplex virus 1 ICP34.5 (Li et al., 2011), or African swine fever virus DPL71 protein (Zhang et al., 2010). Measles virus was shown to inhibit antiviral type I interferon production by targeting PP1, and thus preventing RIG-I like receptor and MDA5 dephosphorylation and activation (Davis et al., 2014). Importantly, targeting the RVxF-pocket of PP1 by using the non-catalytic small molecule inhibitor 1E7-03 was shown to have an antiviral effect against HIV-1 and several RNA viruses (Ammosova et al., 2014, 2018; Baer et al., 2016; Carey et al., 2018; Tigabu et al., 2018), highlighting the compound as a promising broad-spectrum antiviral candidate. Human cytomegalovirus (HCMV), a ds-DNA member of the betaherpesvirus sub-family, is a wide-spread opportunistic pathogen that can cause serious illness in newborns and immunocompromised patients, especially organ transplant recipients (Sweet, 1999). HCMV manipulates protein translation in its favor without inhibiting total protein synthesis, e.g., by UL38-mediated mTORC1 activation (Moorman et al., 2008), and in contrast to most viruses depends on an increased abundance of cellular ribosomes and protein translation factors (McKinney et al., 2014). Metabolic manipulation starts immediately with HCMV entry and the induction of growth factor receptor signaling (Soroceanu et al., 2008). Furthermore, HCMV employs multiple mechanisms to maintain and promote protein translation while coping with cellular energy stress, e.g., by controlling ribosomal initiation and elongation factors, or metabolic signaling pathways such as PI3K and mTOR (Vincent et al., 2016). It is known that HCMV packages human PP1 from the infected host cell into the tegument of its mature viral particles (Michelson et al., 1996). After viral entry, the phosphatase is then released into the newly infected cell (Michelson et al., 1996). The ensuing cellular hypophosphorylation is further sustained by viral upregulation of PP1 in the host cell (McKinney et al., 2014). Similarly, HCMV tegument proteins are also hypophosphorylated (Rieder et al., 2017b). However, the role of PP1 in HCMV infection is still unknown. In this study, we investigated the role of PP1 during HCMV infection by using a small molecule protein–protein interaction inhibitor as a molecular tool of noncatalytic PP1 inhibition.

## Materials and Methods

### Cell Culture

Human foreskin fibroblasts (HFF, kindly supplied by Dr. Thomas Mertens, University Ulm, Germany) between passages 8 and 25, ARPE-19 retinal pigment epithelial cells (ATCC CRL-2302) and HeLa cells (kindly supplied by Prof. Johannes Berger, Medical University of Vienna) were cultured under standard conditions (37°C, 5% CO_2_) in Dulbecco's modified eagle medium (DMEM GlutaMAX, Gibco) supplemented with 10% fetal calf serum (FCS, Gibco) and penicillin–streptomycin mix (Gibco) at a final concentration of 100 U/mL. For PP1 inhibition experiments, cells were pre-treated with 1E7-03 or DMSO for 30 min before infection or mock infection. 1E7-03 was then kept in the medium at a constant concentration throughout the experiment. Media and treatment were resupplied to the cells after 48 h. 1E7-03 (purity above 98%) was synthesized by Enamine LTD (Kiev, Ukraine) as described previously (Ammosova et al., 2014), dissolved in DMSO at a stock solution of 20 mM and stored at –80°C.

### HCMV Infection

HCMV strain AD169 was prepared as described previously (Rieder et al., 2017a). For virus production, HFF were grown to confluence and subsequently infected with HCMV AD169 at a multiplicity of infection (MOI) of 0.01. The viral inoculum was replaced by culture medium after 90 min. Upon visibility of a total cytopathic effect (CPE) (usually 8–10 days post-infection), the viral supernatant was harvested and stored at –80°C. After thawing, cell debris was removed by centrifugation at 3,000 *g* for 20 min. Infectivity of the viral inoculum was then measured using a plaque assay as described elsewhere (Britt, 2010). For functional plaque reduction assays, HFF were seeded into 24-well plates and pre-treated with vehicle (DMSO) or 1E7-03 for 30 min at the indicated concentrations. The cells were then incubated with viral inoculum at 60 plaque forming units per well for 90 min, maintaining a constant concentration of DMSO or 1E7-03 through all phases of the experiment. Ganciclovir (Merck Millipore) was added at the indicated concentrations after removal of the inoculum. The cells were then overlaid with growth medium containing 0.5% low-melting point agarose. After solidification of the agarose layer, the plates were incubated for 10 days at 37°C in a CO_2_ incubator, then fixed with 2% formaldehyde and analyzed for number of plaques after staining with 0.02% methylene blue. Plaque numbers were normalized to the mean of DMSO-treated control samples. IC50 values were calculated with Graphpad Prism (dose–response inhibition). For infection of cell cultures with HCMV strains AD169 or TB40, cells were seeded into 6-well plates, starved overnight in FBS-free growth medium, and pre-treated with the indicated compounds. Viral inoculum was added to the cells at an MOI of 1, if not indicated otherwise, incubated for 1 h at 37°C and then replaced with fresh growth medium including 2% FBS.

For virion purification, supernatant of infected cells was first pre-cleared by centrifugation for 20 min at 3,000 *g*, 16°C. The viral suspension was then ultracentrifuged over a 20% sucrose (w/v) cushion at 70,000 *g* for 40 min at 16°C. Virus pellets were resuspended in urea lysis buffer (10 mM Tris, 4 M urea, 100 mM NaH_2_PO_4_, pH 7.4, 1% Halt protease and phosphatase inhibitor) and sonicated for 4 × 10 s. Protein concentration was measured using a Pierce 660 nm assay before addition of Laemmli loading dye and SDS-PAGE.

### RNA Isolation and RT-qPCR

Total RNA was extracted from cells using the PureLink RNA mini kit (Thermofisher Scientific) according to the manufacturer's instructions, including a 15-min on-column DNA digest using an RNAse-free DNase kit (Qiagen). First strand cDNA was synthesized using iScript reverse transcriptase (Biorad), which includes a mix of oligo-dT and random hexamer primers. Quantitative reverse transcription PCR (qRT-PCR) was performed using a StepOne Plus Real-Time PCR System (Applied Biosystems) and Applied Biosystems' Power SYBR Green Master Mix. Sequence-specific oligonucleotide primers were designed using Primer3Plus (Untergasser et al., 2007) or obtained from RTprimer-DB (Pattyn et al., 2003) or PrimerBank (Spandidos et al., 2010) and synthesized by Microsynth (Austria). Relative expression values were normalized to human GAPDH (glyceraldehyde-3-phosphate dehydrogenase) using the comparative threshold cycle method [2–ΔCt (Schmittgen et al., 2008)], or 2–ΔΔCt (Livak and Schmittgen, 2001) for comparison of mRNA expression between samples (e.g., infected vs. mock-infected). Primers used are described in Supplementary Table 1.

### SDS-PAGE and Western Blot

For protein harvest of whole cell lysates, cells were washed with PBS and lysed in lysis buffer (50 mM Tris pH 7.5, 500 mM NaCl, 1% NP-40, 0.1% SDS) supplemented with 1% Halt protease and phosphatase inhibitor (Thermofisher). Protein concentration of lysates was determined using the Pierce BCA protein assay kit (Thermofisher). Equal amounts (10–30 μg) of sample were loaded onto self-cast 6/8% polyacrylamide gels or 4–15% precast Mini Protean TGX polyacrylamide gradient gels (Biorad) and subjected to SDS-PAGE. Proteins were blotted to a 0.45 μm PVDF membrane (Thermofisher) and subsequently stained with Ponceau S solution for total protein visualization. Destained membranes were blocked with StartingBlock TBS blocking buffer (Thermofisher) and subsequently probed with primary antibody overnight at 4°C. The monoclonal antibodies p-AMPKα T172 #50081, AMPKα #5831, AMPKβ1 #12063, b-actin #8457, p-EEF2 T56 #2331, p-EEF2K S366 #3691, p-eIF2α S51 #9721, p-eIF4e S2019 #9741, eIF4e #2067, p-Raptor S792 #2083, Raptor #2280, RXRα 3085, p-ULK1 S555 #5869, ULK1 #8054, and secondary goat α-Rabbit HRP #7074 were obtained from Cell Signaling Technology. The monoclonal antibodies eEF2K sc-390710, EEF1G sc-393378, EEF2 sc-166415, EF-Tu (EEF1A) sc-393924, GAPDH sc-47724, HCMV pp65 sc-56976, pp72 sc-69834, pp72/86 sc-69748, pp86 sc-69835, eIF2α sc-133132, PP1 (E-9) sc-7482, PP1α sc-271762, PP1β sc-373782, and PP1γ sc-515943 were from Santa Cruz Biotechnologies. Secondary goat anti-mouse HRP antibody was from Biorad (1706516). Isoform specificity of antibodies was validated elsewhere (Hiraga et al., 2017). The UL32/pp150 IgG2b monoclonal antibody was synthesized from hybridoma cell lines and stems from mice immunized with the XP1 antigen expressed in *E. coli* as described previously (Hensel et al., 1995).

### Phos-TAG SDS-PAGE

For phosphorylation-specific separation of proteins, pre-cast SuperSep Phos-Tag 7.5% (50μM) gels (Wako Fujifilm) were used for SDS-PAGE. Gels were run at constant current of 15 mA for 3 h, then washed 6 × 10 min in Tris/Glycine buffer containing 10 mM EDTA and equilibrated for 2 × 10 min in Tris/Glycine buffer containing 1 mM EDTA. Proteins were blotted to a PVDF membrane overnight at 24 V.

### Co-immunoprecipitation

For co-immunoprecipitation (Co-IP) of AMPK and PP1, HeLa cells were transfected with a total of 25μg pcDNA-His-PP1-H248K and pcDNA-AMPKβ1 using the Turbofect lipofection reagent (Thermofisher) and Optimem serum-free medium (Gibco). Cells were lysed 48 h after transfection (50 mM Tris pH 7.5, 500 mM NaCl, 1% NP-40, 0.1% SDS, and freshly supplemented protease inhibitor cocktail), spinned for 10 min at 10,000 *g* and immediately diluted with 1x TBS after taking aliquots for input and protein concentration measurement using the Pierce BCA assay. Co-IP was performed by the addition of 2μg of the respective antibody (6x-His Tag HIS.H8 MA1-21315 from Invitrogen, control IgG from Santa Cruz Biotechnology or AMPKβ1 from Cell Signaling) for 1 h, followed by the addition of Protein A/G Plus beads (Santa Cruz Biotechnology) for another 2.5 h. Beads were then washed multiple times over spin columns, followed by elution by boiling in SDS-PAGE loading dye for 5 min. Light-chain-specific secondary HRP-conjugated antibodies (Santa Cruz Biotechnology) were used for the ensuing Western Blot detection.

### Nano-LC-FT/MS Analysis

LC-FT/MS analysis was performed on a LC-20AD nano-HPLC system (Shimadzu Corporation, Columbia, MD, USA) coupled to a LTQ XL Orbitrap mass spectrometer (Thermofisher) running with Xcalibur software (version 2.0.7, Thermofisher). Processed peptides or phosphopeptides were resuspended in 50 μL water with 0.1% formic acid (v/v). 10 μL of sample was loaded and analyzed using an in-house C18-packed analytical column (25 cm × 150 μm, 5 μm, 200 Å, Michrom Bioresources, Auburn, CA, USA). Mobile phase A was 0.1% formic acid in water, while mobile phase B was 0.1% formic acid in acetonitrile. Samples were separated with a linear gradient of 6–55 min, 2–40% B, 55–62 min, 40–80% B, 62–70 min, and 80% B (v/v) at the flow rate of 600 nL/min. The Orbitrap was operated under data-dependent acquisition mode. Spray voltage, capillary temperature, and capillary voltage were set to 2.0 kV, 200°C, and 39.5 V, respectively. Full-scan mass spectra were acquired in Orbitrap over 300–2,000 m/z with a resolution of 30,000, followed by MSn scans by CID activation mode. The three most intense ions were selected for fragmentation using collision-induced dissociation (CID) in the LTQ.

### Proteomics

Proteins (150 μg per group) extracted from DMSO or 1E7-03-treated HFF cells infected with HCMV were ultrasonically suspended in sodium phosphate buffer (pH 8.0), reduced in 10 mM dithiothreitol (1 h at 60°C), alkylated with 30 mM iodoacetamide (20 min, room temperature in the dark), and digested with 10 μg trypsin (Promega) at 37°C on an orbital shaker overnight. Peptide mixtures from each group were divided into two equal parts. One half was directly purified by Pierce Graphite Spin Columns (88302, Thermofisher) following the manufacturer's instructions and analyzed by LC-FT/MS. Label-free quantitative analysis was performed on Proteome Discoverer 2.4 (PD 2.4) using Sequest search engine (Thermo Fisher Scientific), against the Uniprot Human database at a false discovery cut off ≤ 1%. Filter settings to define peptide confidence were as follows: charge 2 = 1.5 (XCorr score), charge 3 = 2.0, and charges >4 = 2.5 for high confidence peptides; charge 2 = 0.5, charge 3 = 0.8, and charges >4 = 1 for modest confidence peptides. The other half of peptide mixtures were enriched by high-select TiO_2_ phosphopeptide enrichment kit (88301, Thermofisher). TiO_2_ spin columns were prepared by washing with 20 μL of 0.4% TFA in 80% acetonitrile and 20 μL of 25% lactic acid. Peptide mixtures were dried, resuspended in 150 μL of 25% lactic acid, added to TiO_2_ spin columns, and incubated for 2 x 10 min with end-over-end rotation. Samples were washed with 20 μL of 25% lactic acid and 20 μL of 25% lactic acid twice. Then samples were eluted using 50 μL of 1.5% ammonium hydroxide and 50 μL of 5% pyrrolidine. The elution fractions were combined, acidified by adding 100 μL 2.5% TFA, and purified using graphite spin columns. The enriched phosphopeptides were then submitted to LC-FT/MS analysis. LC-FT/MS raw data were searched by PD 2.4 using the Sequest search engine (Thermofisher), against the Human Uniprot database at a false discovery cutoff ≤ 1%. A maximum of two missed cleavage sites was allowed. The mass tolerance for the precursor ion was set on 30 ppm and for the fragment on 0.1 Da. Phosphorylation of serine, threonine, and tyrosine was enabled as dynamic modifications, while carbamidometylation of cysteine was set as fixed modification. The same filter settings as above were used to determine the peptide confidence. The label-free quantification of phosphopeptides was performed with SIEVE 2.1 software (Thermofisher). Principal component analysis and volcano plots were exported from PD 2.4. and adapted graphically. The mass spectrometry proteomics data have been deposited to the ProteomeXchange Consortium via the PRIDE repository with the dataset identifier PXD023598.

### Protein Array

The Proteome Profiler Human Phospho-Kinase Array Kit (R&D Systems) was used according to the manufacturer's instructions. The array membranes were incubated with 300μg cell lysate overnight. The detected signals were quantified using ImageLab software (BioRad). The signal of the negative control was subtracted from each spot, and values were normalized to the reference spots in each membrane.

### Translation Assays

Protein synthesis was assayed in black-wall 96-well plates using O-propargyl-puromycin (OPP) labeling for 30 min at 37°C followed by Click-iT chemistry using a Click-iT Plus OPP Alexa Fluor 647 Protein Synthesis Assay Kit (Invitrogen) as detailed by the manufacturer. Cells were pre-treated with 1E7-03, cycloheximide (Santa Cruz Biotechnology), or DMSO for 30 min before OPP addition. In HCMV samples, infection was let to proceed for 4 h in the presence of DMSO or 1E7-03 before the addition of OPP. Fluorescence was quantified using a Varioskan plate reader with excitation/emission bands of 650/670 nm for AF647 and 350/451 nm for NuclearMask Blue Fluorescence, respectively. Background fluorescence from the no OPP condition was subtracted from all values. AF647 fluorescence was normalized to the NuclearMask signal to control for cell number.

### Mammalian-2-Hybrid Assay

Mammalian-2-hybrid luciferase assays were performed according to the protocol of the MatchmakerTM system (Clontech) using the pRF-Luc reporter plasmid (Stratagene) for detection of protein–protein interaction. HeLa cells were transfected in 24-well plates using 2 μl Turbofect (Thermofisher) and 850 ng DNA per well according to the manufacturer's instructions. The following plasmid combinations were transfected: 0.1 μg of luciferase reporter plasmid pFR-Luc (Stratagene) and 0.05 μg of pCMV-β-Gal (P204, Promega) for normalization, combined with 0.35 μg of bait (pM, GAL4DBD encoding) and 0.35 μg of prey (pVP16, VP16AD encoding) plasmids (cloning vectors kindly supplied by Markus Kunze, Medical University of Vienna). After 48 h, transfected cells were washed with PBS and lysed in 100μl/well 1x Cell Culture Lysis Reagent (Luciferase Assay System; Promega). Cell lysates were centrifuged at 13,000 rpm at 4°C for 20 min, and the supernatant was tested for luciferase and β-galactosidase activity. For luminescence measurements, luciferase activity was tested using a Varioskan Lux microplate reader (Thermofisher) by well-loop addition of 50μl substrate (Luciferase Assay Reagent; Promega) to 10μl lysate and immediate detection of luminescence. β-Galactosidase content was tested by incubation of cell lysates with 2-Nitrophenyl-β-D-galactopyranoside (ONPG; Sigma) at 37°C and measurement of absorbance at 420 nm in a microplate reader. Luminescence values were normalized to β-galactosidase to account for transfection efficiency (Kunze et al., 2015). Expression of bait and prey proteins was controlled for by verifying GFP fluorescence of pM-bait-eGFP transfected cells and bait/prey protein detection via western blot.

### siRNA Transfections

EEF2k-targeting siRNAs, AMPK-targeting siRNAs, and scrambled control siRNA siCTRL (Hait et al., 2006; Ashour et al., 2014; Li et al., 2014; Bae et al., 2019) were produced by Microsynth (Austria) with 3′ dTdT overhangs, and PP1-specific siRNA mixes were obtained from Santa Cruz Biotechnology (see Supplementary Table 2). For transfections, HFF were seeded into 6-well plates and transfected at 80% confluence with a mixture of 50 nM siRNA and 5 μl Turbofect (Thermofisher) diluted in serum-free Optimem medium (Thermofisher). Five hours after transfection, cells were washed and the medium was replaced with fresh growth medium. After 24 h, cells were transfected a second time. Another 24 h later, HFF were infected with HCMV AD169 and harvested at 24 h post-infection for analysis of viral protein expression by western blot.

### Phosphatase Assays

For *in vitro* dephosphorylation assays, whole cell lysates were incubated with bacterial Lambda phosphatase (λ) or protein phosphatase 1 (New England BioLabs) according to the manufacturer, with a maximum of 4U (PP1) or 5,00 U (λ) for 50 μg protein. Enzymatic reactions were stopped after 30 min at 30°C by adding reducing sample buffer and boiling for 5 min. All sample conditions contained a protease inhibitor cocktail (Sigma-Aldrich); a protease/phosphatase inhibitor mix (Halt, Thermfisher) was used as an additional control.

### Statistical Analyses

If not indicated otherwise, bar graphs and error bars show the mean ± standard error of the mean of 3 or more independent experiments. We analyzed the data using Graphpad Prism 7 using the respective statistical analysis and *post hoc* test indicated in the figure legends. An alpha value of 0.05 was considered significant (^*^p < 0.05, ^**^p < 0.01, ^***^p < 0.001, and ^****^p < 0.0001).

## Results

### The HCMV Virion Is Enriched in Human PP1 and Contains Multiple PP1 Interacting Proteins

**Figure 1:**
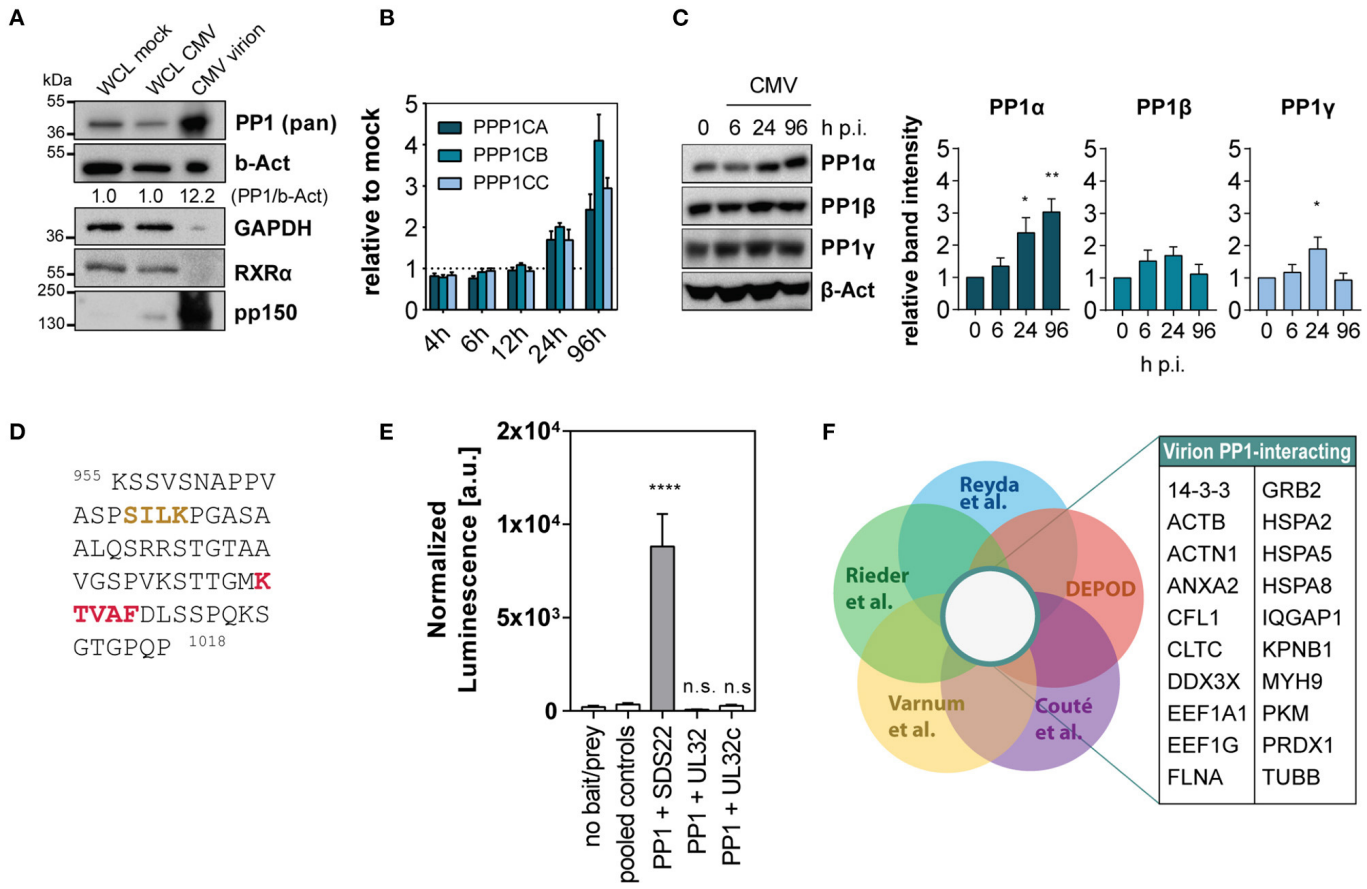
Human PP1 binding proteins in the HCMV virion. **(A)** Immunoblots showing abundance of the indicated proteins in whole cell lysate (WCL) from mock-infected and HCMV-infected cells at 96 h p.i. compared to HCMV virion lysate from ultra-centrifuged HCMV AD169. 15 μg total protein was loaded in each lane. **(B)** Expression of PP1 isoform mRNA shown as fold change compared to mock at the indicated time points post-infection with HCMV AD169 at MOI 3. Representative of 3 independent experiments. **(C)** Western blots showing PP1 isoform specific staining (Hiraga et al., 2017) of mock or HCMV AD169 infected cells (MOI 3) at the indicated time points post-infection. **(D)** Sequence of HCMV UL32 protein C terminus containing SILK (yellow) and RVxF (red) motifs. **(E)** Mammalian-2-hybrid testing protein–protein interaction of PP1 and SDS22 (positive control), UL32, or a c-terminal variant of UL32 (UL32c) containing the SILK and RVxF motif. In the no bait/prey negative control, only the luciferase and beta-galactosidase vectors were transfected. For simplicity, all negative bait/prey empty vector control combinations are shown as “pooled controls.” Asterisks indicate significant differences from Dunnett's ANOVA *post hoc* test compared to pooled controls. **(F)** Venn diagrams showing the gene name list of intersection between the indicated MS Datasets (Varnum et al., 2004; Reyda et al., 2014; Rieder et al., 2017b; Couté et al., 2020) and the PP1 interactor list from DEPOD (PP1-PPi) (Li et al., 2013).

Despite the reported presence of the PP1 catalytic subunit in the HCMV tegument, it is not known how the phosphatase is incorporated into the virion or what is the purpose for the virus to carry it despite tight spatial restriction. Since the substrate specificity of PP1 as a holoenzyme greatly depends on its specific regulatory subunit, we sought to gain insight into the potential composition of the virion-derived PP1 holoenzyme. In fact, we were able to detect all three isoforms, PP1α, PP1β and PP1γ, in virus lysate from HCMV AD169 virions after ultracentrifugation using western blot (Supplementary Figure 1A). While it has been discussed that other macromolecules such as human mRNA present in the virion may merely represent their abundance at the packaging site (Terhune et al., 2004), we observed an enrichment of PP1 in cell-free HCMV lysate: PP1 was present at a higher concentration in virus lysate compared to whole cell lysate of mock-infected or HCMV-infected fibroblasts, in contrast to beta-actin which is also part of the HCMV virion according to multiple studies (Varnum et al., 2004; Reyda et al., 2014; Rieder et al., 2017b). The retinoid X receptor (RXRα) and GAPDH, used as cellular controls abundant in whole cell lysate, were absent from the virion (Figure 1A). HCMV-imported PP1 is hypothesized to cause cellular hypophosphorylation shortly after HCMV attachment (Michelson et al., 1996), and in addition PP1 was reported to be upregulated during HCMV infection (Hakki and Geballe, 2008). In an *in vitro* infection model using the laboratory strain AD169, we confirmed PP1 upregulation at the level of mRNA in the course of infection (Figure 1B). However, only the PP1α protein isoform, but not PP1β and PP1γ, was consistently and significantly increased during infection as determined by isoform-specific immunoblots (Figure 1C). The PP1 holoenzyme usually involves binding of a regulatory subunit to one or multiple binding pockets via docking on the regulatory protein such as the RVxF, SpiDoc, SILK, IDoHA, or MyPhonE motifs, with the RVxF motif being the most common one occurring in most of the PP1 regulatory proteins and also in the few known viral PP1 binding proteins (Nekhai et al., 2007; Zhang et al., 2010; Li et al., 2011). Thus, as a next step we tested the possibility of direct binding of HCMV tegument proteins to PP1. Of the 38 HCMV proteins that are described to occur in the tegument (reviewed in Kalejta, 2008), 10 contain at least one classical RVxF motif, most of which are not conserved in a closely related betaherpesvirus (Supplementary Figure 1B). Other common linear PP1-docking motifs on HCMV tegument proteins are absent except for a “SILK” motif in the C-terminus of UL32 (Figure 1D). We tested protein–protein interaction between PP1 and RVxF-containing viral tegument proteins using a mammalian-2-hybrid assay; yet we were unable to detect any direct binding of PP1 to UL32 (Figure 1E) and other RVxF-containing tegument proteins (Supplementary Figure 1C) in this system compared to the positive control SDS22. Thus, we next considered the possibility that PP1 is incorporated into the tegument as a holoenzyme bound to another human protein. Mass spectrometry (MS) analyses of purified HCMV virions from our group and others further indicate that in addition to PP1, HCMV integrates multiple other human proteins into its viral particle. From these data, we have identified 20 human proteins inside the virion that were reported as hits in 4 independent MS datasets (Varnum et al., 2004; Reyda et al., 2014; Rieder et al., 2017b; Couté et al., 2020) and that are also listed in the PP1 interactome according to the DEPOD database (Li et al., 2013) (Figure 1F). This list also contains members of the ribosomal translation elongation factor complex and 14-3-3 proteins, which were validated by western blot (Supplementary Figure 1D).

### PP1 Inhibition Is Detrimental for HCMV Replication

**Figure 2:**
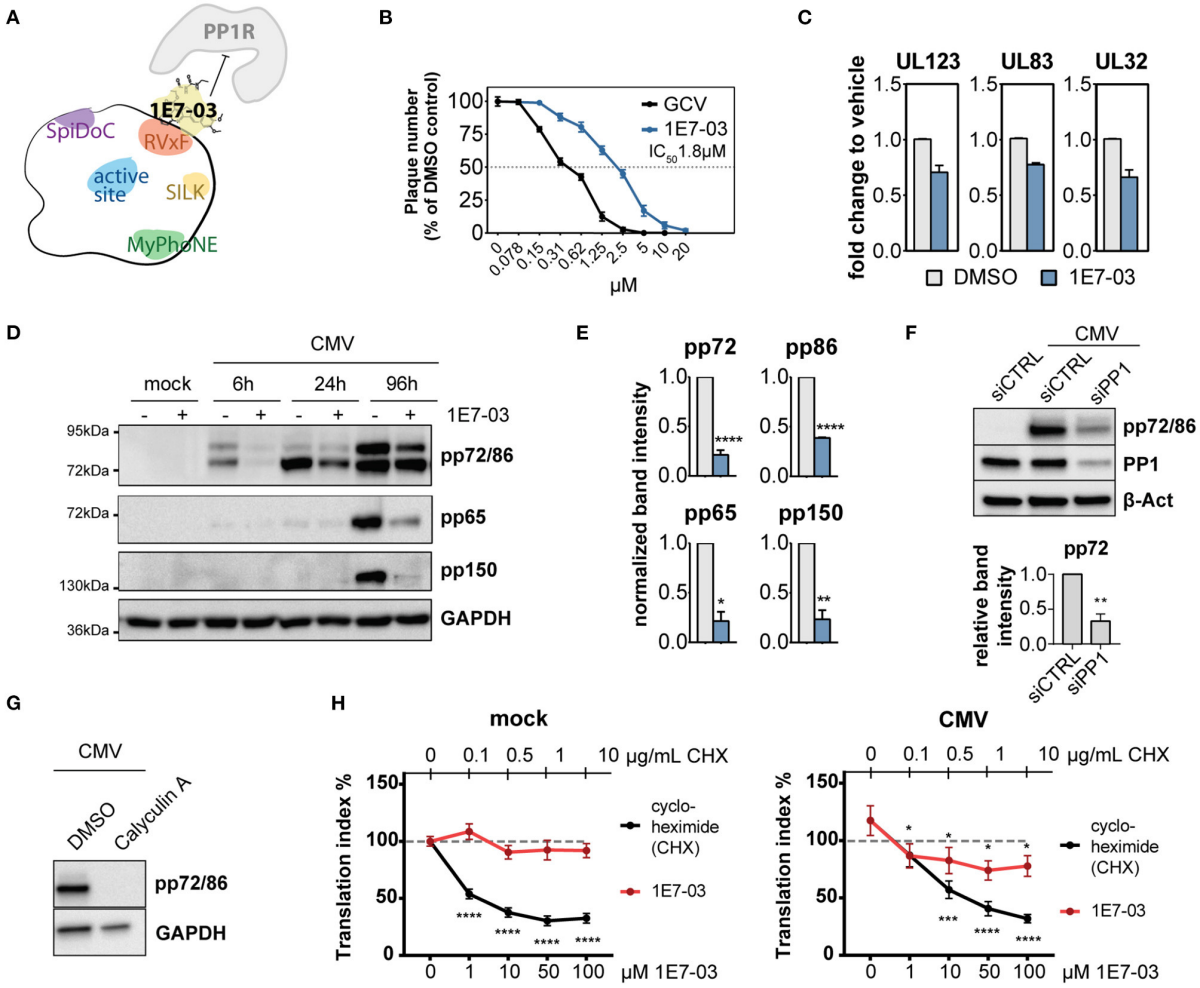
Expression of PP1 and influence of PP1 inhibition in human cytomegalovirus (HCMV) infection. **(A)** Cartoon of PP1 and its most common docking motifs with exemplary binding of a PP1 regulatory protein (PP1R) inhibited by 1E7-03. **(B)** Plaque reduction assay of human foreskin fibroblasts (HFF) infected with HCMV AD169 and treated with 1E7-03 or ganciclovir; percentage compared to DMSO control (*n* = 4). **(C)** Viral mRNA expression was measured in HCMV-infected HFF by RT-qPCR 6 h p.i. (UL123) or 96 h p.i. (UL83 and UL32), showing fold-change of the indicated cDNAs compared to infected cells treated with vehicle only (mean ± SEM). **(D)** Expression of HCMV proteins in HFF treated with DMSO (–) or 20μM 1E7-03 (+) harvested at the indicated time points were measured by western blot. **(E)** Bar graphs show mean and SEM of relative band intensity normalized to GAPDH from 3 to 6 independent experiments as shown in **(C)** (6 h p.i. for pp72 and pp86 or 96 h p.i. for pp65 and pp150). Asterisks indicate significant differences from unpaired Student's *t*-tests between DMSO and 1E7-03 treatment. **(F)** HFF were transfected with non-targeting control siRNA or PP1-targeting siRNA twice and subsequently infected with HCMV. Cell lysates were harvested 24 h p.i. and stained for immediate early proteins (representative blot and quantification of band intensities relative to beta-actin from 3 experiments). **(G)** Western blot showing expression of HCMV immediate early proteins in HFF treated with DMSO or 1 nM calyculin A at 6 h p.i. **(H)** ClickIT O-propargyl-puromycin (OPP) assay showing the translational index (AF647 fluorescence normalized to cell count) in mock (top) or HCMV-infected (bottom) cells at 4 h p.i. 100% (dashed line) indicates the baseline activity of untreated wells in mock cells. Asterisks indicate significant differences to the untreated control (2-way ANOVA with Dunnett's *post hoc* test).

Given the hypothesized importance of PP1 for the virus during infection, we next sought to test the effect of PP1 inhibition on viral propagation. Catalytic PP1 inhibition is toxic to cells already at low molecular levels and unspecific since most PP1 inhibitors also co-target PP2a (Swingle et al., 2007; Munday, 2013). In order to study PP1 function during HCMV infection, we used 1E7-03, a non-catalytic small molecule inhibitor of PP1 that targets the regulatory “RVxF” -binding pocket of PP1 (see Figure 2A) and that was previously shown to have antiviral activity against HIV-1 and several other RNA viruses (Ammosova et al., 2014, 2018; Baer et al., 2016; Carey et al., 2018; Tigabu et al., 2018). 1E7-03 significantly inhibited HCMV replication in a plaque reduction assay at an average IC50 of 1.8 μM±0.12 (Figure 2B). As previously reported, 1E7-03 has a low toxicity profile (Ammosova et al., 2014), making it possible to study the cells over longer periods of time. While 1E7-03-treated and mock-infected or HCMV-infected cells showed limited cell death, Calyculin A treated cells died upon prolonged time in culture, even in the absence of HCMV infection (Supplementary Figures 2A–C). Importantly, 1E7-03 treatment did not seem to alter PP1 expression during infection compared to the vehicle control (Supplementary Figure 2D). In order to investigate the mechanism behind 1E7-03-mediated inhibition of HCMV replication, we next tested whether viral transcription and translation of immediate early, early, and late proteins were affected by treatment with 1E7-03. As reported in Figure 2C and Supplementary Figure 2E, mRNA transcription of both immediate early and late genes surprisingly did not significantly change at the time points measured, indicating that viral entry and transcription *per se* are likely not affected by 1E7-03. However, viral translation was severely attenuated by the treatment, including representatives of immediate early (pp72, pp86), early (pp65), and late (pp150) proteins throughout the HCMV life cycle, apparent already at the immediate-early expression stage of the HCMV life cycle (Figures 2D,E). We observed a similar reduction of viral immediate early proteins when PP1 was transiently knocked down with specific siRNAs (Figure 2F). The catalytic phosphatase inhibitor Calyculin A also reduced HCMV protein expression (Figure 2G), but HFF showed poor viability upon prolonged time in culture (Supplementary Figures 2A–C). We also observed that starting 1E7-03 treatment later during infection led to a less pronounced downregulation of viral proteins (Supplementary Figure 2F) as well as reduced effectivity in plaque reduction assays (Supplementary Figure 2G). Importantly, a similar effect of 1E7-03 on CMV immediate early protein expression could be observed in retinal pigment epithelial cells infected with the TB40/E CMV strain (Supplementary Figure 2H). Moreover, the reduction of HCMV protein expression by 1E7-03 was dose dependent (Supplementary Figure 2I). These results suggest that 1E7-03 affects a stage of the HCMV replication cycle that starts before the onset of viral DNA replication, and is able to inhibit the replication HCMV at a low micromolar range.

### 1E7-03 Attenuates Mitogenic Signaling Pathways During Infection

**Figure 3:**
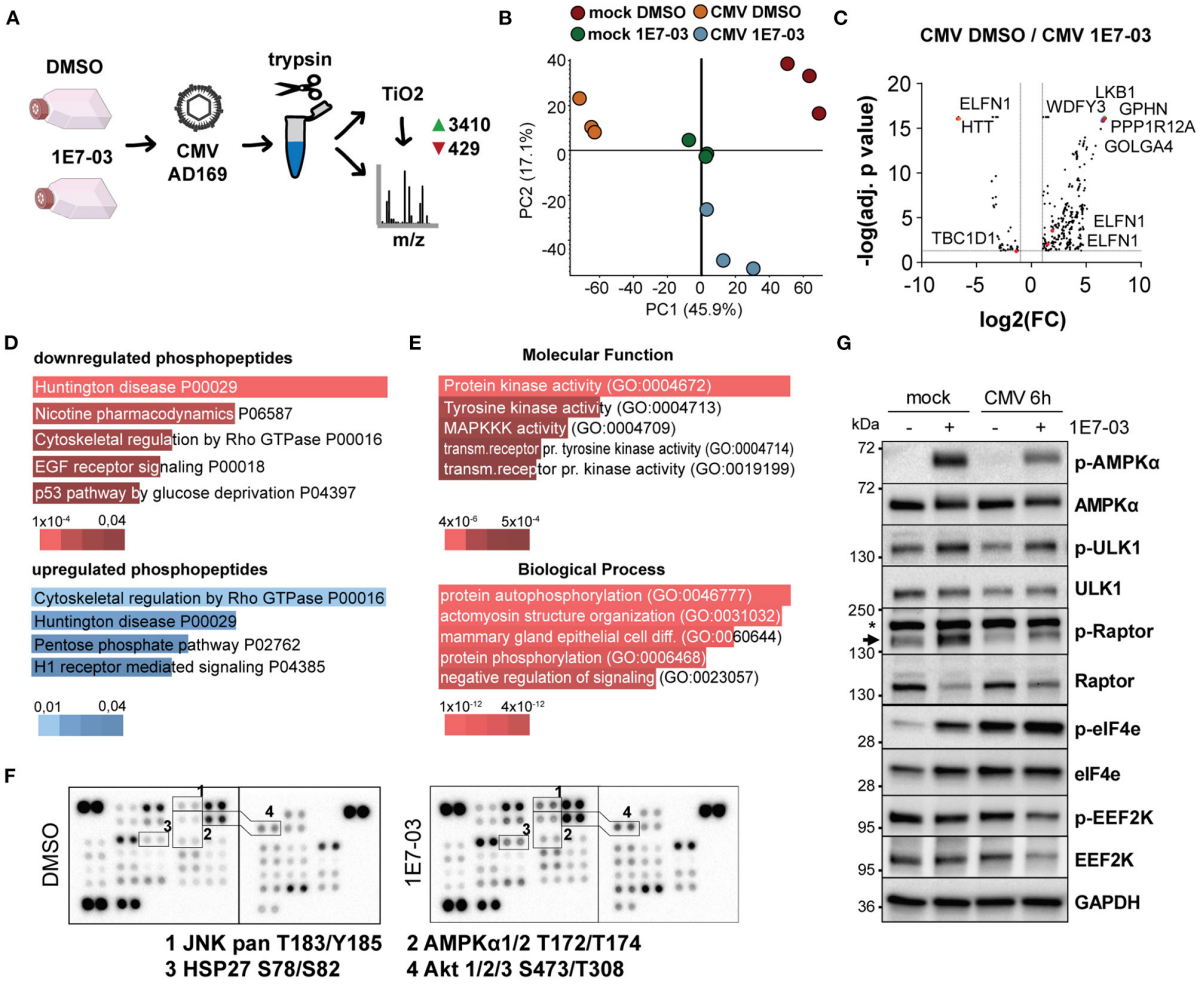
Phosphoproteomic analysis of 1E7-03 treatment. **(A)** Mass spectrometry workflow of 1E7-03 treatment and human cytomegalovirus (HCMV) infection for phosphoproteome analysis resulting in the identification of 3410 upregulated and 429 downregulated peptides between DMSO and 1E7-03 treatment at 2 h p.i. (>1.5-fold change, adj. *p* < 0.05). **(B)** Principal component analysis of TiO_2_-enriched sample triplicates of mock and HCMV-infected cells 2 h post-infection. **(C)** Volcano plot showing differences in differentially regulated phosphopeptides in DMSO-treated vs. 1E7-03 treated human foreskin fibroblasts (HFF) infected with HCMV. Highlighted dots show peptides from PRKAA (AMPK) targets. **(D)** Panther pathway enrichment analysis using EnrichR bar plots (Chen et al., 2013) sorted by *p*-value ranking showing the top 5 hits of downregulated or upregulated phosphopeptides, respectively. Bar length and color (as indicated below) represent the *p*-value of the specific gene-set or term. **(E)** GO term enrichment of differentially expressed phosphopeptides (Enrichr) sorted by *p*-value ranking showing the top 5 hits. **(F)** Phosphokinase protein array of lysates from HCMV-infected HFF harvested 4 h post-infection. **(G)** Immunoblots of 1E7-03 treated HCMV or mock-infected HFF showing AMPK activation (Thr172) and phosphorylation of AMPK targets and inhibitory EEF2K S366 phosphorylation.

Given the observed attenuation of viral translation rates and the role of PP1 in cellular metabolism, we next analyzed global translation rates during infection and PP1 inhibition. In a fluorescence-based puromycin assay, we indeed observed slightly attenuated translation rates with 1E7-03 during infection, but not in uninfected cells (Figure 2H). We next conducted a proteome and TiO_2_-enrichment phosphoproteome analysis of HCMV-infected cells treated with vehicle or 1E7-03 and harvested at 2 h post-infection performed in triplicate. This resulted in 3839 differentially regulated phosphopeptides between the vehicle and 1E7-03 group in HCMV-infected cells (Figures 3A–C and Supplementary Material). A pathway enrichment analysis showed that 1E7-03 induced changes in Rho GTPase-dependent cytoskeletal regulation, metabolic stress, and mitogenic signaling pathways (Figures 3D,E). Similarly, a phosphokinase protein array at immediate early times post-infection indicated changes in PI3K/Akt, JNK, and AMPK mitogenic signaling pathways upon treatment with 1E7-03 (Figure 3F). Specifically, activating phosphorylation of AMPKα and AMPK targets (ULK1, eIF4e, and inhibitory Raptor phosphorylation) was increased by 1E7-03 as determined by phospho-specific western blot (Figure 3G). However, phosphorylation of mTOR seemed not to be affected by 1E7-03 (Supplementary Figure 3).

### PP1 Is an AMPK Phosphatase and Its Inhibition Leads to AMPK and EEF2 Hyperphosphorylation

**Figure 4:**
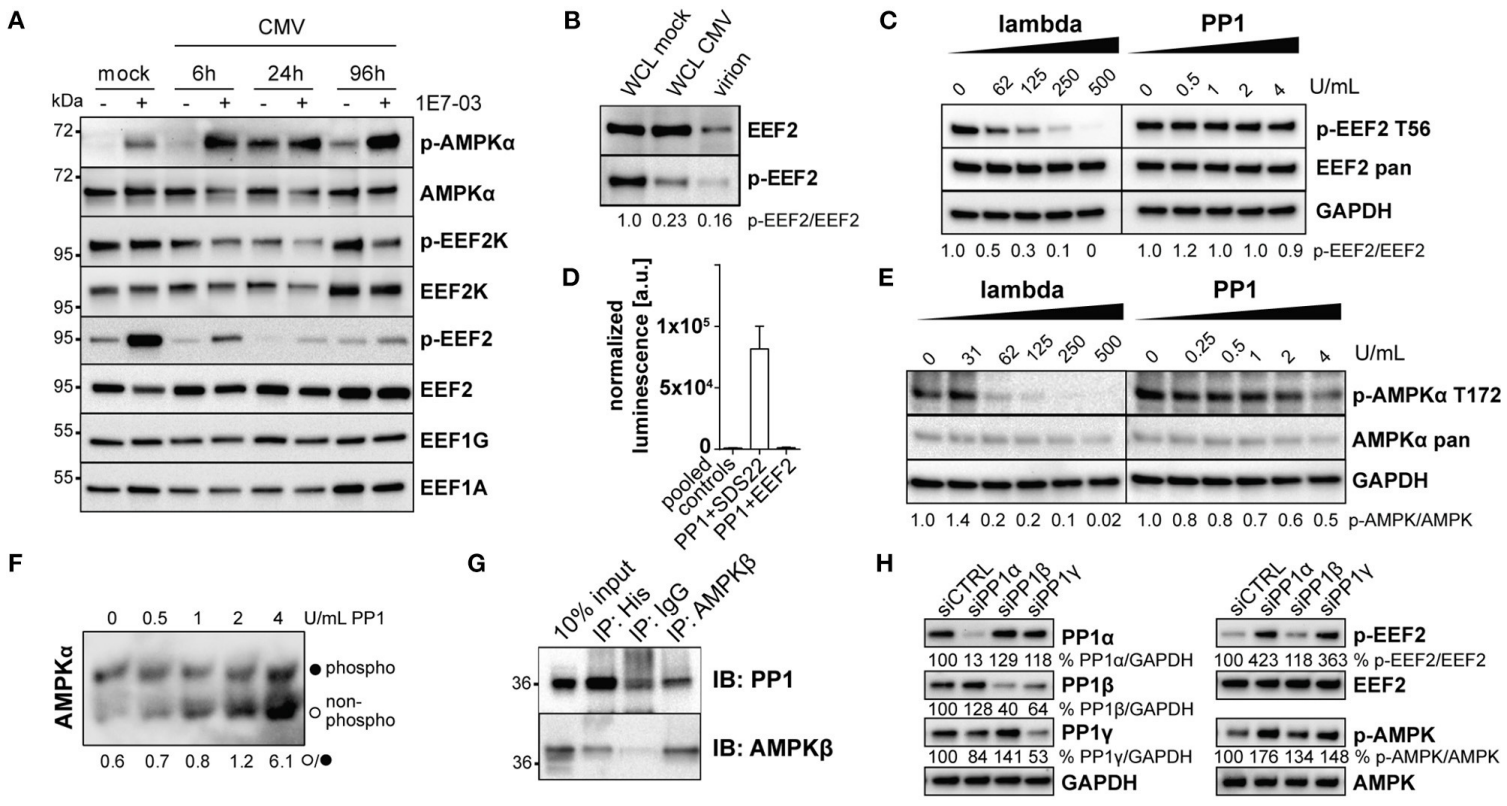
PP1 targets AMPK. **(A)** Immunoblots showing AMPK and translation elongation factor phosphorylation during human cytomegalovirus (HCMV) infection under treatment of DMSO (–) or 1E7-03 (+). **(B)** Immunoblots of EEF2 in whole cell lysate of mock- or HCMV infected human foreskin fibroblasts (HFF) and cell-free virus lysate (20μg protein/lane). **(C)** *in vitro* dephosphorylation assay of lysates from FBS and glucose-starved HFF incubated with the indicated amounts of λ or PP1 phosphatase. Representative images of p-EEF2 T56-specific western blot are shown. **(D)** Mammalian-2-hybrid assay of pVP16-PP1 bait and pM-EEF2 or positive control pM-SDS22 prey showing luminescence normalized to beta-galactosidase. For simplicity, all negative empty vector control combinations are shown as “pooled controls.” **(E)** *in vitro* dephosphorylation assay of lysates from FBS and glucose-starved HFF incubated with the indicated amounts of λ or PP1 phosphatase. Representative images of p-AMPKα T172-specific western blot are shown. **(F)** Lysates from FBS and glucose-starved HFF were incubated with the indicated amounts of PP1 phosphatase *in vitro* and subsequently separated via Phos-Tag SDS-PAGE. A representative pan-AMPKα-specific immunoblot shows the ratio between unphosphorylated and phosphorylated AMPKα. **(G)** Co-immunoprecipitation of His-PP1 and AMPKβ in lysates of HeLa cells overexpressing a pcDNA-His-PP1 H248K catalytic inactive substrate trap mutant and pcDNA-AMPKβ1. **(H)** Representative western blot showing siRNA-mediated knockdown of PP1 isoforms of HFF harvested 24 h post-CMV infection.

During viral infection, cellular stress pathways are activated in the cell that normally lead to shutdown of protein translation via phosphorylation of translation initiation factor 2α (eIF-2α), e.g., by protein kinase R. Targeting eIF-2α for dephosphorylation by PP1 can re-enable translation, as done by Herpes simplex virus 1 (Li et al., 2011). Thus, we investigated the possibility that HCMV may employ a similar mechanism which might be inhibited upon treatment with PP1-modulators. However, we detected only minor and transient changes in eIF-2α phosphorylation during HCMV infection upon treatment with 1E7-03 (Supplementary Figure 4A) or downstream ATF-4 upregulation (Supplementary Figure 4B). On the other hand, AMPKα phosphorylation was greatly increased by 1E7-03 throughout the infection. Inhibitory phosphorylation of EEF2K, an AMPK target (Johanns et al., 2017), was decreased by the compound (Figure 4A). Furthermore, inhibitory Threonine-57 phosphorylation of the elongation factor EEF2 was clearly increased throughout the infection upon PP1 inhibition via 1E7-03 (Figure 4A). This is especially noteworthy, since EEF2 is part of the human proteins in the HCMV virion, predominantly its dephosphorylated form (Rieder et al., 2017b and Figure 4B). 1E7-03-induced EEF2 and AMPKα phosphorylation started already at concentrations lower than 20μM close to the IC50 of the antiviral effect (Supplementary Figure 4C). Since EEF2 also contains an RVxF binding motif and is carried in the virion along with PP1, we next investigated whether EEF2 might directly be affected by PP1 inhibition. In order to determine whether EEF2 can be directly phosphorylated by PP1, we performed an *in vitro* phosphatase assay. However, the addition of exogenous PP1 to cellular lysate was not sufficient for EEF2 dephosphorylation (Figure 4C). Also, EEF2 failed to directly interact with PP1 in a mammalian-2-hybrid assay (Figure 4D). Since we hypothesized that EEF2K is activated upon 1E7-03 treatment as a result of AMPK activation (Figures 3A, 4A), we next explored signaling events upstream of EEF2 phosphorylation affected by PP1 inhibition. The AMPKα subunit, which has been identified as a PP1 substrate in mice (Garcia-Haro et al., 2010), could be dephosphorylated by PP1 *in vitro* (Figures 4E,F). In addition, we were able to detect PP1 and AMPKβ in the same complex by Co-IP from HeLa cells overexpressing both a PP1 H248K catalytic mutant and AMPKβ1 (Figure 4G), indicating that PP1 likely targets the AMPK holoenzyme also in human cells. Similar to PP1 inhibition using 1E7-03, also siRNA-mediated knockdown of PP1 led to phosphorylation of AMPK and EEF2 during CMV infection (Figure 4H).

### PP1 Is Necessary for Mitogenic Activation via EEF2 in Infected Cells

**Figure 5:**
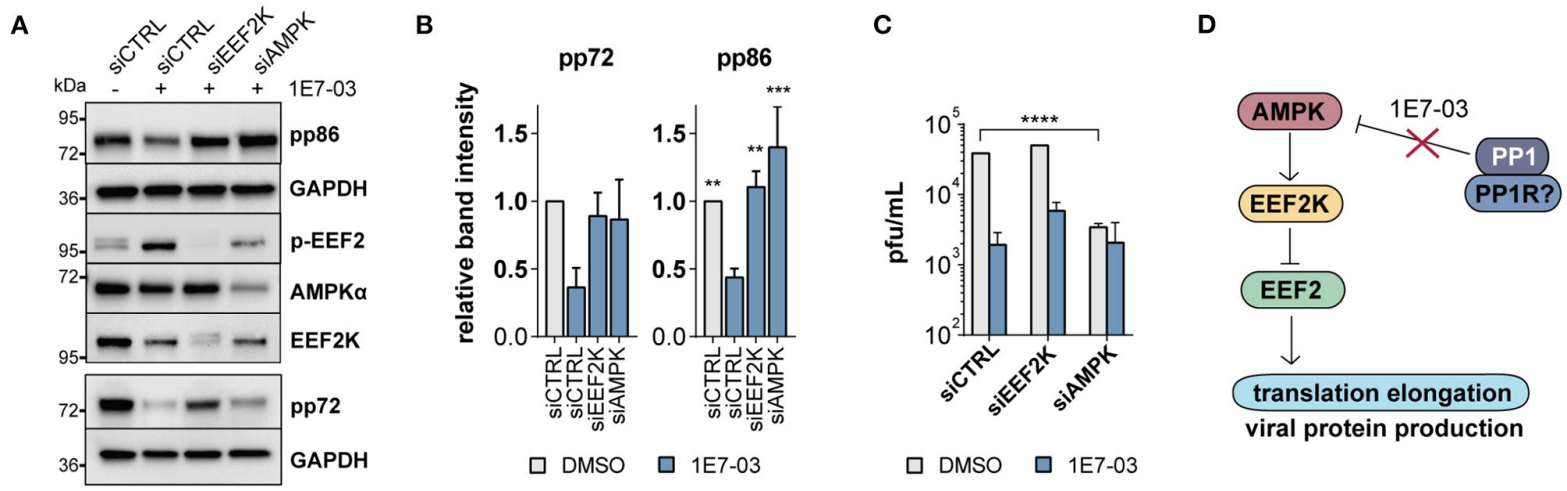
EEF2K knockdown restores viral protein translation. **(A)** Representative western blot showing non-targeting siRNA (siCTRL) compared to EEF2K and AMPK knockdown in cells infected with human cytomegalovirus (HCMV) and treated with 1E7-03 for 24 h. **(B)** Quantification of relative band intensities of viral pp86 and pp72 expression at 24 h p.i. as shown in **(A)** (*n* = 3 for pp86 and *n* = 2 for pp72). Asterisks indicate significant differences to control siRNA treated with 1E7-03 as determined by ANOVA Dunnett's multiple comparison *post hoc* test (*p* = 0.14 for pp72 ANOVA). **(C)** Determination of viral titers of cell supernatants of HFF transfected with siRNAs targeting EEF2K or AMPK, treated with DMSO or 1E7-03, and harvested at 96 h post-infection (representative of *n* = 3). **(D)** Pathway map showing an overview of the proposed mechanism of PP1 metabolic activation via the AMPK/EEF2K/EEF2 axis inhibited by 1E7-03.

To date, EEF2K is the only known kinase for EEF2 T56 phosphorylation. In a knockdown experiment, we therefore attempted to rescue the 1E7-03-induced reduction of viral immediate early proteins by diminishing the accumulation of EEF2 phosphorylation using EEF2K-targeting siRNA mixes. In EEF2K-deficient cells, immediate early protein expression was normal despite the presence of 1E7-03 (Figures 5A,B). Similarly, knockdown of the EEF2K inactivating kinase AMPK led to a reduced effect of 1E7-03 immediate early protein attenuation (Figures 5A,B). 1E7-03 reduced HCMV titers in supernatants harvested from treated cells as determined in a plaque assay using fluorescently labeled virus. This effect was partly reversed in cells with siRNA-mediated inhibition of EEF2K (Figure 5C), indicating an important role of PP1 in EEF2K-mediated inhibition of infection. However, knockdown of AMPK led itself to impaired production of viral progeny (Figure 5C), in line with previous reports (Terry et al., 2012). A tight and timely regulation of AMPK is likely crucial for HCMV infection since both chemical AMPK activation and inhibition are known to inhibit HCMV propagation (Kudchodkar et al., 2007; Terry et al., 2012; Dunn et al., 2020). In our proposed model, PP1 inhibition by 1E7-03 leads to early AMPK activation and EEF2 inactivation, followed by attenuated translation and inhibition of lytic HCMV infection (Figure 5D).

## Discussion

HCMV produces asymmetric enveloped virions with an extensive tegument layer. The release of the tegument into the cell provides an instant supply of proteins upon lytic infection. As multiple studies have evaluated by now using proteomic techniques, the viral particle contains not only viral, but also many human proteins, many of which are robustly detected across multiple datasets (Varnum et al., 2004; Reyda et al., 2014; Rieder et al., 2017b; Couté et al., 2020). Also the human phosphatase PP1 has been previously described to be part of the tegument layer (Michelson et al., 1996). Here, we show that PP1 is enriched in the virion compared to cellular lysates, implying an incorporation in a directed manner, although we did not discern the role of dense bodies in our experimental setup, and contamination with cellular microvesicles cannot be excluded. However, PP1 has also been detected in highly purified virions (Couté et al., 2020). PP1 specificity depends on the interaction with a regulatory subunit, thus we originally hypothesized that an HCMV tegument protein functions as a PP1 regulatory subunit for incorporation into the virion. When we screened HCMV tegument proteins for potential PP1 binding motifs, the pp150 (UL32) protein stood out containing both an RVxF and a SILK motif. However, we could not confirm an interaction between PP1 and UL32 and several other tegument proteins containing RVxF motifs (Figure 1). We thus hypothesize that PP1 is incorporated as a human holoenzyme from the cell, but cannot exclude a role of other viral proteins. While our experimental setup cannot discern between a role of virus-derived vs. cellular PP1 inhibition, we found an essential role of PP1 for HCMV protein translation and replication using the novel PP1 inhibitor 1E7-03 (Figure 2). In contrast to catalytic PP1 inhibition, which mostly co-inhibits PP2a and is highly toxic to cells (Swingle et al., 2007; Munday, 2013), 1E7-03 caused no increased cell death (Supplementary Figure 2), presumably because it does not inhibit the catalytic groove and cannot actively break up existing PP1-PP1R interactions (Lin et al., 2017). This highlights 1E7-03 as a molecular tool for PP1 loss of function experiments over longer periods of time. Importantly, 1E7-03 inhibited HCMV at similar effective concentrations that are necessary to specifically inhibit protein–protein interactions between PP1 and RVXF-harboring regulatory proteins (Lin et al., 2019). From a stoichiometric point of view, the amount of virion-derived PP1 is supposably insignificant compared to the abundance of cellular phosphatases, and the majority of 1E7-03-targeted PP1 is likely cellular. However, virion-imported human kinases have been shown to specifically contribute to cellular target protein phosphorylation (Nogalski et al., 2007). It will therefore be necessary to further investigate the composition of PP1 holoenzymes in the viral vs. host environment to elucidate the specific function of virus-derived PP1. Manipulation of host metabolism and the cellular stress response starts already at pre-immediate early times with binding and entry of the virus. HCMV manipulates metabolic signaling pathways in a way to uphold features that are beneficial to the virus while inhibiting detrimental effects (Shenk et al., 2014). PP1 inhibition using 1E7-03 led to disturbance of metabolic signaling already at 2–6 h post-infection. In a phosphoproteomic analysis and a phosphokinase array, many of the 1E7-03-affected signaling pathways were related to cellular stress and metabolic activity (Figure 3). PP1 inhibition even led to an attenuation of global protein translation during HCMV infection (Figure 2H). We identified changes in AMPKα and EEF2 phosphorylation as a possible mechanism of 1E7-03-mediated translation inhibition. AMPK not only regulates fatty acid and glucose metabolism but also modulates protein synthesis and cell growth through the EEF2K/EEF2 and TSC2/mTOR pathways (Johanns et al., 2017). EEF2 itself, which catalyzes the ribosomal movement along the mRNA, is also present in the HCMV virion in its active unphosphorylated form (Figure 4B), but it remains to be determined whether virus-imported EEF2 is of biological relevance. While EEF2 T56 phosphorylation could not be dephosphorylated by PP1 in HFF, we identified PP1 as a potential AMPKα phosphatase (Figures 4E–G), so far only described in mice (Garcia-Haro et al., 2010). siRNA-mediated knockdown of EEF2 kinase or AMPKα attenuated the 1E7-03 mediated effect of viral protein reduction (Figure 5). EEF2K knockdown also showed a trend to reconstitute viral titers late in infection, but this was not statistically significant. AMPK knockdown by itself reduced HCMV titers as already reported by others (Terry et al., 2012). It needs to be added that modulation of a promiscuous phosphatase such as PP1 likely induces many more changes in the cell, AMPK phosphorylation being only one of them.

In the literature, AMPK has been described to play an ambivalent role during HCMV infection, since both its inhibition and activation have been reported to be detrimental for HCMV replication. HCMV infection has been shown to modestly increase total AMPK but not change the amount of phosphorylated AMPKα (Terry et al., 2012). Inhibition of AMPK or its kinase CAMKK2 is known to block the glycolytic activation of the cell, thereby decreasing HCMV viral progeny (McArdle et al., 2012; Terry et al., 2012). On the other hand, the AMPK activating molecule AICAR was also reported to inhibit HCMV immediate early expression (Kudchodkar et al., 2007). In addition, it has been suggested that during very early times of infection, AMPK dephosphorylation might be promoted (Kudchodkar et al., 2007). Our study provides evidence that this dephosphorylation might be mediated by PP1 and suggests a novel layer of AMPK regulation by inhibition of the AMPK/EEF2K/EEF2 axis via PP1 starting very early in infection (Figure 5D). A better understanding of the impact of virus-host interactions on cellular phosphorylation and antiviral responses is of high clinical relevance for the identification of potential broad-spectrum antiviral targets and drug development. Lastly, this study might help shed light on the therapeutic potential of targeting Ser/Thr phosphatases, which are considered challenging drug targets.

## Data Availability Statement

The mass spectrometry proteomics data have been deposited to the ProteomeXchange Consortium via the PRIDE partner repository with the dataset identifier PXD023598.

## Author Contributions

CStec designed the study, performed experiments and data analysis, created the graphs, and wrote the manuscript. AK, LM-H, SM, and FR-R performed experiments and data analysis. M-TK prepared experiments and provided technical assistance. XL performed experiments and contributed to proteomic data analysis. SN contributed essential materials and guidance. CStei designed and supervised the study. All authors critically revised the manuscript.

## Conflict of Interest

A patent application on 1E7-03 activity against HCMV has been filed by Howard University with SN and CStei as co-inventors based on parts of the data in this publication. The remaining authors declare that the research was conducted in the absence of any commercial or financial relationships that could be construed as a potential conflict of interest.
