# Supplementary material for: Protein Phosphatase 1 Regulates Human Cytomegalovirus Protein Translation by Restraining AMPK Signaling
Source: Front Microbiol. 2021 Jul 15;12:698603. doi: 10.3389/fmicb.2021.698603 (PMC8320725; doi:10.3389/fmicb.2021.698603)
Supplement: Supplementary file 1 [file Data_Sheet_1.docx]

Supplementary Material

# Supplementary Figures and Tables

## Supplementary Figures


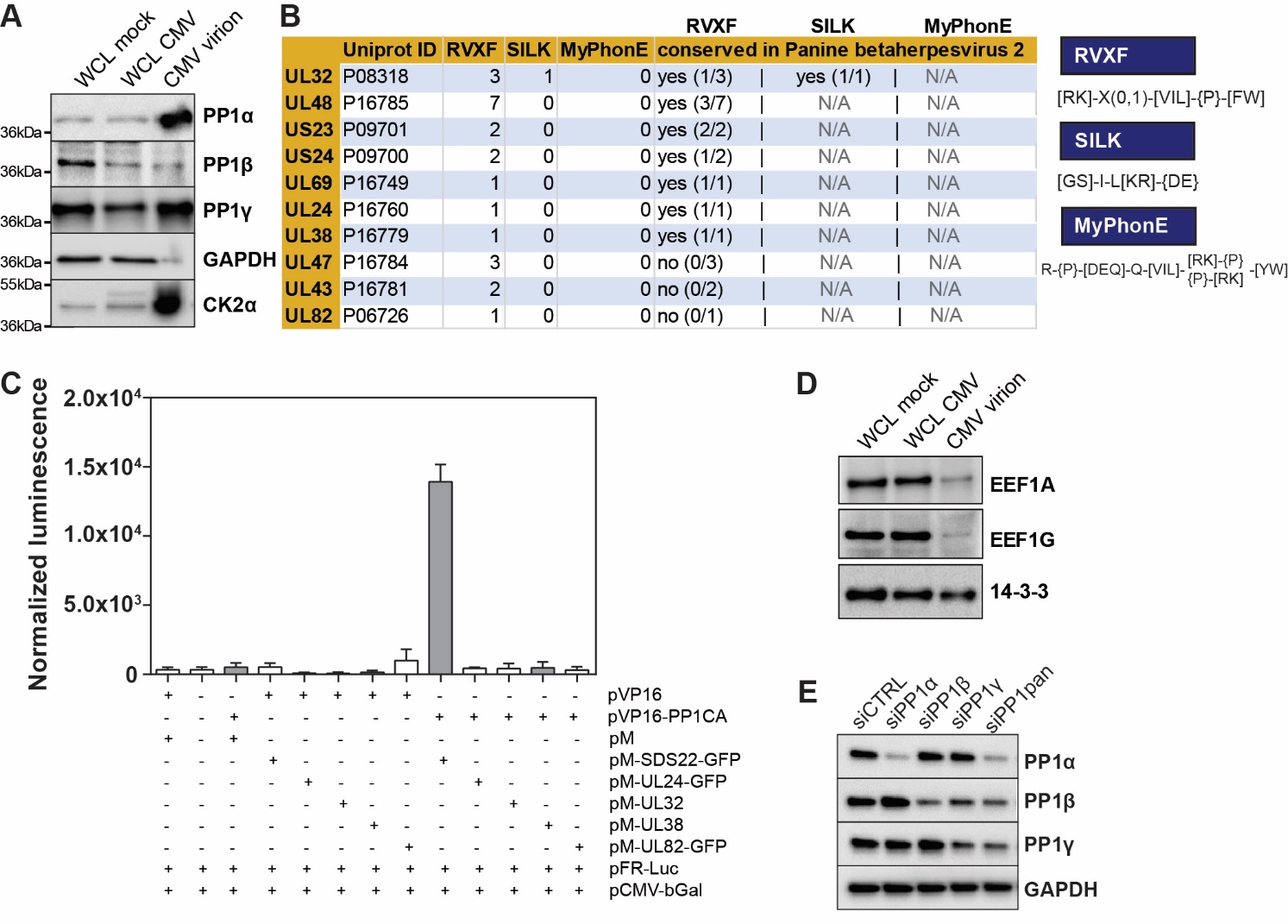


**Supplementary Figure 1.** PP1 in the HCMV tegument. **(A)** Immunoblots showing abundance of the indicated proteins in whole cell lysate (WCL) from mock and HCMV infected cells at 96 hours p.i. compared to HCMV virion lysate from ultra-centrifuged HCMV AD169 (15μg protein per lane). See antibody specificity in (E). **(B)** Table showing HCMV tegument proteins which contain PP1 binding motifs and their conservation in Panine betaherpesvirus 2. **(C)** Mammalian2hybrid assay testing protein-protein interaction of PP1 fused to the VP16 transactivation domain with SDS22 (positive control), UL24, UL32, UL38 or UL82 proteins fused to the pM DNA binding domain (n=2). **(D)** Immunoblots of human proteins in whole cell lysate and cell-free virus lysate (20μg protein/lane). **(E)** PP1 isoforms were knocked down twice in HFF using specific siRNA mixes. Western Blots show isoform specific antibody detection of the same PP1 antibodies used in (A); cells were harvested 24 hours after the second transfection.


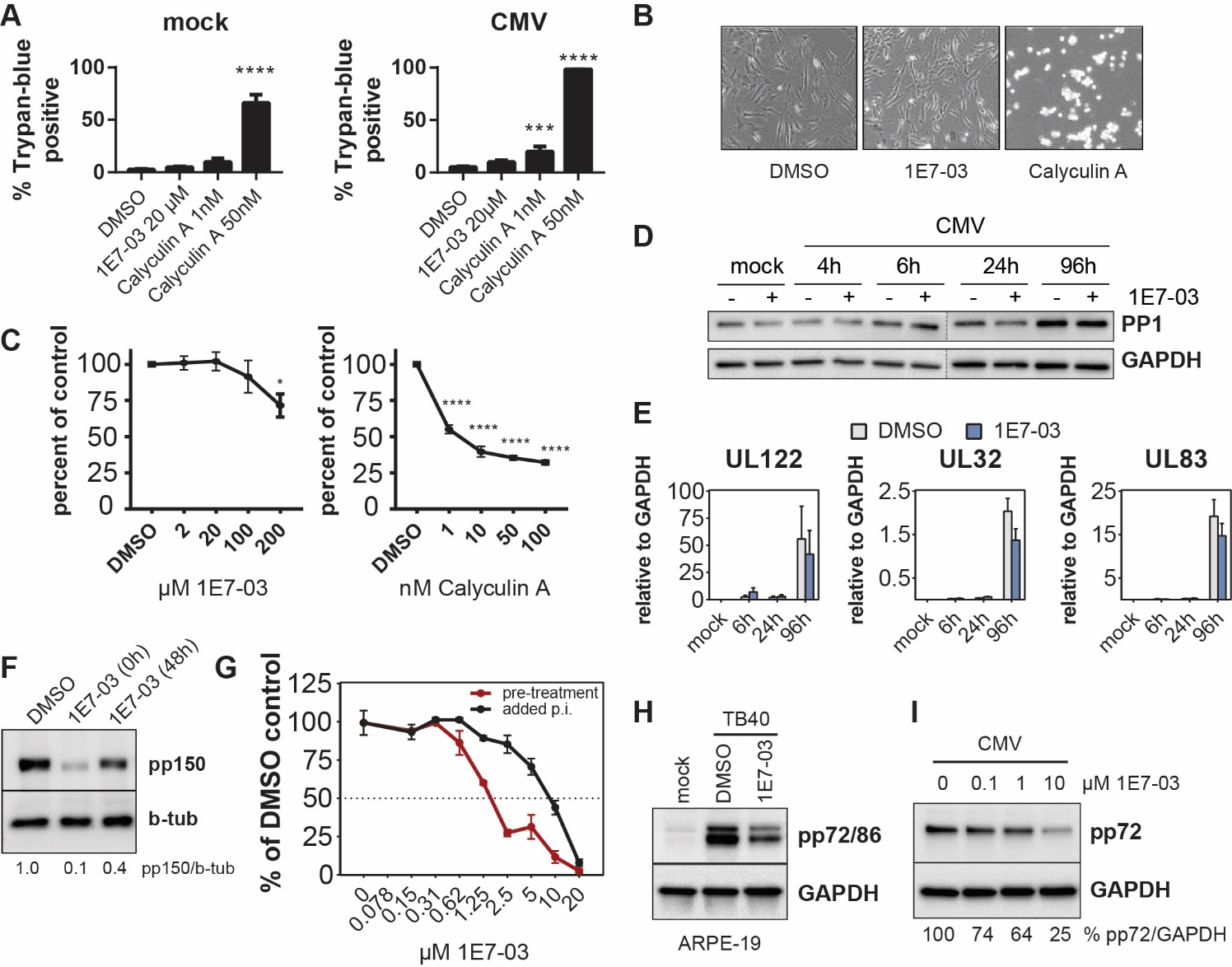


**Supplementary Figure 2.** Effects of 1E7-03. **(A)** Cell death assay of HFF harvested 24h p.i. showing the percentage of trypan blue positive cells of total cells counted after treatment with DMSO or the indicated concentration of 1E7-03 or the PP1/PP2a inhibitor Calyculin A (1way ANOVA and Tukey's post hoc test compared to DMSO control). **(B)** Representative bright field images of HFF infected with HCMV AD169 at 24 hours post infection with treatment of the indicated compounds. **(C)** Viability assay measuring fluorescence after 1h Alamar Blue incubation after 48 hours of treatment with the indicated compounds (1way ANOVA and Tukey's post hoc test compared to DMSO control). **(D)** PP1 expression in DMSO (-) or 1E7-03 (+) treated cells determined by Western Blot. **(E)** Time course of viral mRNA expression in HFF treated with DMSO or 10uM 1E7-03. **(F)** pp150 (UL32) immunoblots from AD169-infected HFF treated with DMSO, treated with 1E7-03 starting before infection (0h) or 2 days after infection (48h). **(G)** Plaque assay of HFF infected with CMV AD169 and treated with 1E7-03, either added 0.5h before infection (“pre-treatment) or added after 1 hour of virus inoculation (“added p.i.”); percentage compared to DMSO control. **(H)** Western Blot of ARPE-19 cells harvested 24 hours post infection with the TB40/E strain. **(I)** CMV-infected HFF were treated with DMSO (0) or the indicated amounts of 1E7-03 and harvested 6 hours post infection (representative of n=2).


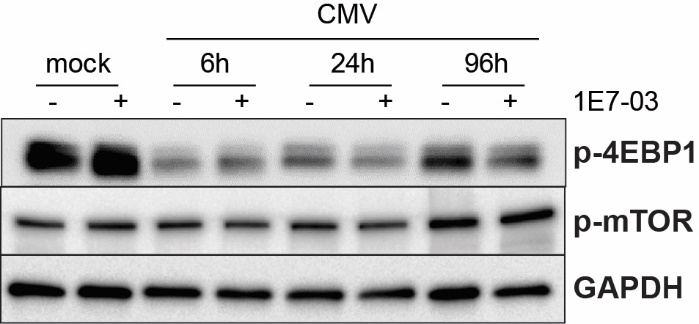


**Supplementary Figure 3.** Western Blot of HCMV-infected HFF harvested at the indicated time points. Antibodies specific to mTOR phosphorylation on Ser2448 and 4EBP1 phosphorylation of Thr37/46 were used, respectively.


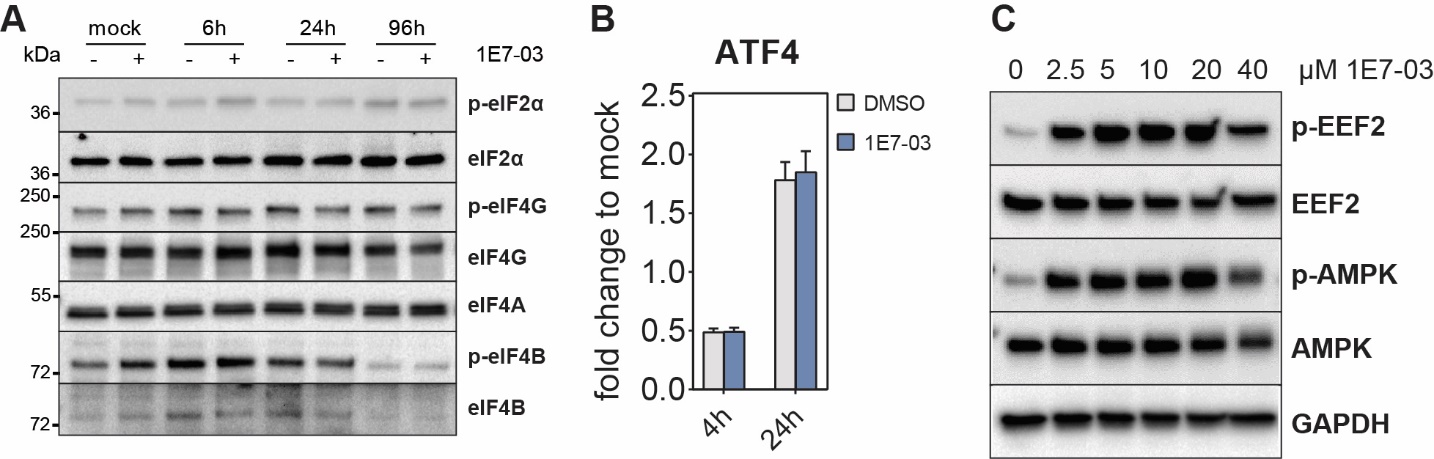


**Supplementary Figure 4.** Phosphorylation of translation initiation factors. **(A)** Immunoblots of p-eIF2α (Ser51), total eIF2α and members of the eIF4 complex in lysates harvested at the indicated time points of CMV infection, treated with DMSO (-) or 20μM 1E7-03 (+). **(B)** ATF4 expression was measured in infected HFF by qPCR 4h and 24h p.i., showing fold-change compared to mock-infected cells treated equally. Samples were normalized to GAPDH as a reference gene. **(C)** Western Blot of HCMV-infected HFF harvested 6h p.i. and treated with the indicated amounts of 1E7-03.


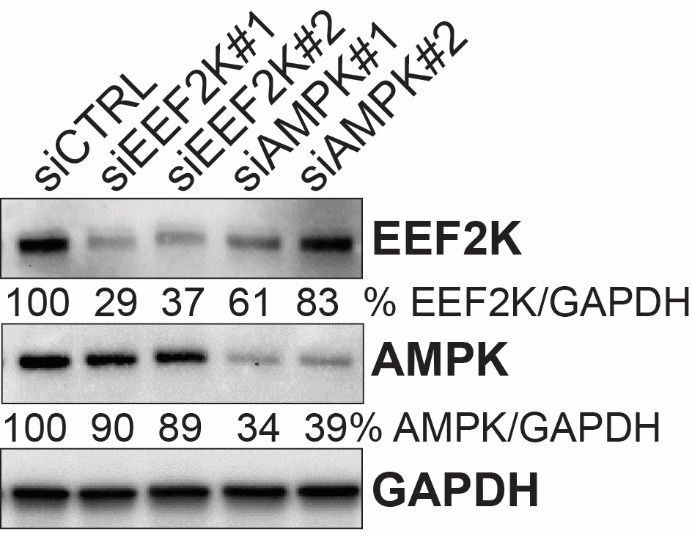


**Supplementary Figure 5.** Transfection of single siRNAs as indicated in Supplementary Table 2. HFF were transfected twice with 40nM of the indicated siRNAs. Immunoblots of EEF2K and AMPK knockdowns harvested 24 hours after the second transfection are shown.

## Supplementary Tables

Supplementary Table 1

| Targeted gene | Forward | Reverse |
| --- | --- | --- |
| PPP1CA | 5’-AAGTACCCCGAGAACTTCTTCC-3’ | 5’-GTAGAAACCATAGATGCGGTTGA-3’ |
| PPP1CB | 5’-CGAGTTTGATAATGCTGGTGGA-3’ | 5’-CGGCGGATTAGCTGTTCGAG-3’ |
| PPP1CC | 5’-CTCAACATCGACAGCATTATCCA-3’ | 5’-CACGAGACTTTAAGCACAGTCC-3’ |
| IE1 (UL123) | 5’-GAAATTCACTGGCGCCTTTA-3’ | 5’- CCCGCTTATCCTCAGGTACA-3’ |
| pp65 (UL83) | 5’- CATCAACGTGCACCACTACC-3’ | 5’- GCTCTTTCCACTGGTTCTGC-3’ |
| pp150 (UL32) | 5’- ATCACGGATACCGAGACGAG-3’ | 5’- GCCAGCAGGAACGTTAACTC-3’ |
| GAPDH | 5’- AAGGTGAAGGTCGGAGTCAAC-3’ | 5’- GGGGTCATTGATGGCAACAAT-3’ |
| ATF4 | 5’- GACGGAGCGCTTTCCTCTT-3’ | 5’- TCCACAAAATGGACGCTCAC-3’ |

Supplementary Table 2

| Targeted mRNA | siRNA sequence |
| --- | --- |
| EEF2K (#1) | 5’-GCCAACCAGUACUACCAAA-3’ |
| EEF2K (#2) | 5’-AAGCUCGAACCAGAAUGUC-3 |
| AMPK (#1) | 5’-AUGAUGUCAGAUGGUGAAUUU-3’ |
| AMPK (#2) | 5’-AAUGGAAUAUGUGUCUGGAGG-3’ |
| control | 5’-AAUUCUCCGAACGUGUCACGU-3’ |
| PP1alpha | sc-36299 Santa Cruz Biotechnology, proprietary mix of 3-5 specific siRNAs 19-25nt long |
| PP1beta | sc-36295 Santa Cruz Biotechnology, proprietary mix of 3-5 specific siRNAs 19-25nt long |
| PP1gamma | sc-36297 Santa Cruz Biotechnology, proprietary mix of 3-5 specific siRNAs 19-25nt long |
| PP1 | Equimolar mix of sc-36299, sc-36295 and sc-36297 |
